# Effects of Microplastic (MP) Exposure at Environmentally Relevant Doses on the Structure, Function, and Transcriptome of the Kidney in Mice

**DOI:** 10.3390/molecules28207104

**Published:** 2023-10-15

**Authors:** Ting Shen, Wenjing Zhang, Yirun Wang, Haizhu Li, Jingwei Wu, Qian Wang, Li Qin, Lu Zhang, Cuiqing Liu, Ran Li

**Affiliations:** School of Public Health, Joint China-US Research Center for Environment and Pulmonary Diseases, Zhejiang International Science and Technology Cooperation Base of Air Pollution and Health, Zhejiang Chinese Medical University, Hangzhou 310053, China201812213603015@zcmu.edu.cn (J.W.);

**Keywords:** microplastics, mice, nephrotoxicity, transcriptome

## Abstract

As a common emerging environmental pollutant, microplastics (MPs) have been detected in a variety of environmental media and human bodies. The potential toxic effects and mechanisms of MPs need to be revealed urgently. MPs can be deposited in the kidney, and exposure to high doses of MPs can cause nephrotoxicity in experimental animals. In this study, we investigated the effects of exposure to polystyrene microplastics (PS-MPs) at environmentally relevant doses (0.1 and 1 mg/L) on kidney structure, function, and transcriptome in mice. We found that mice exposed to PS-MPs in drinking water for eight weeks had no change in body weight or kidney coefficient. PS-MPs administration decreased the levels of blood urea nitrogen (BUN) in mice, while serum creatinine (CRE) and uric acid (UA) concentrations were unaffected. Through using periodic acid–Schiff (PAS) and Masson staining, we discovered that the glomerular tuft area increased in the PS-MP-treated mice, while the degree of renal fibrosis remained unchanged. Furthermore, renal cortex transcriptomic analysis identified 388 and 303 differentially expressed genes (DEGs) in the 0.1 and 1 mg/L dose groups, respectively. The DEGs were highly enriched in mitochondrial-related terms and pathways of thermogenesis and oxidative phosphorylation. Moreover, protein–protein interaction (PPI) network analysis revealed that cytochrome b-c1 complex subunit 10 (UQCR11) and cytochrome c oxidase subunit 3 (MT-CO3) were important node proteins. These findings suggest that environmental exposure to MPs can cause abnormalities in renal structure and filtration function and that long-term exposure to MPs may be a risk factor for renal disease.

## 1. Introduction

Plastics are widely used due to their good ductility, durability, and low production costs. The global production of plastics has expanded dramatically over the last few decades, rising from 1.5 million metric tons in 1950 to 390.7 million metric tons in 2021 [1], and further increases in their production and use are expected. However, plastics can be broken into small fragments via physicochemical degradation and subsequently become microplastics (MPs), a term which refers to fibers, films, fragments, or granular particles smaller than 5 mm in size [2,3]. In addition to the degradation of larger plastics, MPs also include the plastic microbeads in personal care products, the plastic pellets used in industrial manufacturing, and the plastic fibers used in synthetic textiles, etc. [2,4]. MPs have a variety of chemical compositions, including polystyrene (PS), polyvinyl chloride (PVC), polyethylene (PE), polypropylene (PP), and polyethylene terephthalate (PET), etc. [2].

In 2014, Law and Thompson first reported the presence and distribution of MPs in the ocean [5]. Subsequent environmental investigations found that MPs are ubiquitous and that they exist in large quantities in freshwater [6], tap water [7], soil [8], the atmosphere [9], and even polar glaciers [10]. Therefore, it is not surprising that MPs have been detected in many organisms, especially aquatic animals [11,12]. It is important to note that humans may also be exposed to MPs by ingesting contaminated water and food, inhaling contaminated air, etc. [13,14]. Ingested MPs are able to cross the intestinal epithelium and enter other tissues through the bloodstream [15]. Some studies have reported the discovery of MPs in human blood [16] and organs, including the liver, kidney, intestines, lungs, and placenta [17,18,19,20]. Hence, the potential toxicity and health hazards of MPs deserve further attention.

The kidney is an important excretory organ that removes waste products and excess fluid from the body. Previous studies have documented the bioaccumulation of MPs with varying particle sizes in the kidneys of mice after oral administration [21]. Although a few studies have found that MPs could induce histopathological and functional abnormalities regarding the kidney following gastrointestinal exposure, the results are inconsistent [22,23,24,25]. For example, Wang et al. discovered that polystyrene microplastics (PS-MPs) could lead to lower renal coefficients and higher levels of serum creatinine (CRE) and blood urea nitrogen (BUN) in rats [23]. However, another study showed that serum CRE decreased in mice after MP treatment, while BUN was not affected [22]. Xiong et al. reported the vacuolar and granular degeneration of renal tubular epithelial cells and tubular atrophy in mice kidneys after exposure to MPs [25], while no obvious damage in kidney tissues was presented in Mu et al.’s study [24]. These disparities could mainly be attributed to varying MP exposure dosages. In actuality, the majority of existing reports describe evaluations of the renal toxicity of MPs at high exposure concentrations, whereas the effects of MPs at environmentally relevant doses on the kidney have rarely been reported. Moreover, the toxic mechanisms of action of MPs on the kidneys of mammals are not yet understood.

PS-MPs are the prevailing type of MPs found in the environment, and they are widely utilized in toxicological research [12,22,23,26]. In this study, we explored the effects of PS-MPs exposure at environmentally relevant doses on renal structure and function using a mouse model. We exposed the mice to MPs through their drinking water. In addition, the transcriptome of the renal cortex was analyzed to investigate the putative renal toxicity mechanism of MPs. This work will help to increase our understanding of the nephrotoxic effects of MPs in mammals at environmentally relevant doses and shed light on their probable toxic mechanism.

## 2. Results

### 2.1. Effects of PS-MP Exposure on Weight Gain and Kidney Coefficient and Function

**Figure 1:**
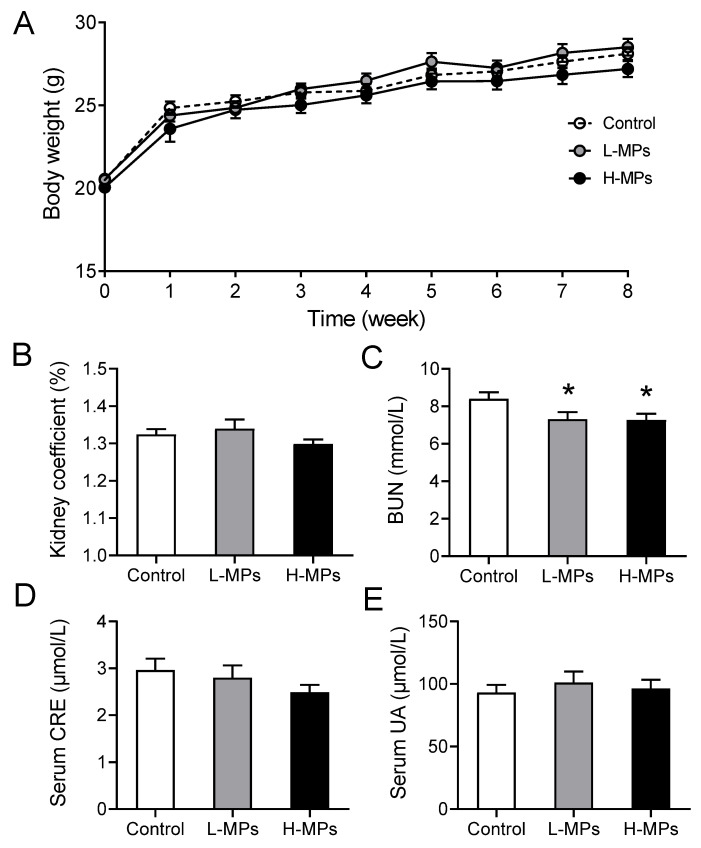
Effects of PS-MPs on body weight, kidney coefficient, BUN, serum CRE, and serum UA. Mice were given sterilized drinking water with 0, 100, or 1000 μg/L of PS-MPs for 8 weeks. Body weight (**A**), kidney coefficient (**B**), BUN (**C**), serum CRE (**D**), and serum UA (**E**) were measured. Data are expressed as means ± SEM; *n* = 8. * *p* < 0.05 compared with control group. SEM: standard error of the mean.

The body weight of each individual mouse was measured every week, and PS-MP exposure exerted no effect on body weight during the 8-week exposure period (Figure 1A). At the end of the exposure period, the kidney coefficients were calculated, and no significant change was observed among mice from different groups (Figure 1B). However, both the low-dose (L-MPs) group and the high-dose (H-MPs) group had considerably lower BUN levels than the control group (Figure 1C). In addition, serum CRE and serum UA concentrations were not affected by PS-MPs exposure (Figure 1D,E). These findings suggest that exposure to PS-MPs at environmentally relevant doses has the potential to impact renal function.

### 2.2. Effects of PS-MP Exposure on Kidney Histopathology

**Figure 2:**
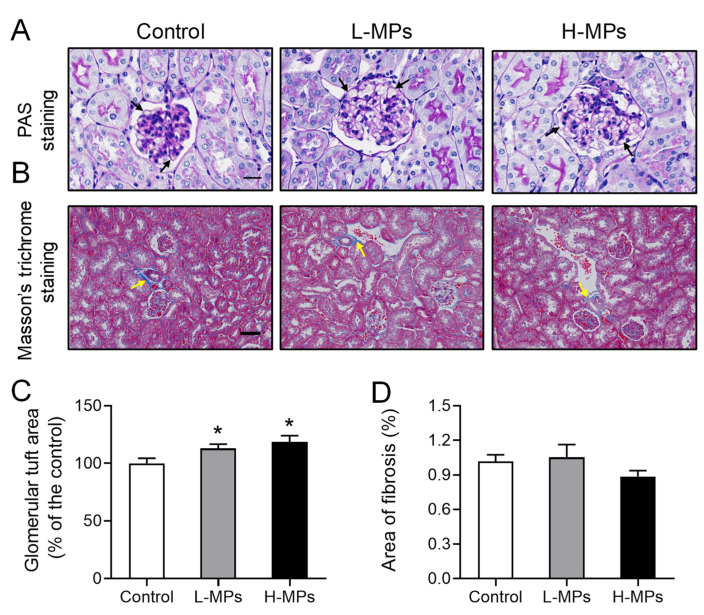
Effects of PS-MPs on kidney histopathology. Representative images of mice kidneys after PAS staining ((**A**), scale bar: 20 μm) and Masson’s trichrome staining ((**B**), Scale bar: 50 μm). Quantification of glomerular tuft area (**C**) and collagen fibers (**D**) in mice from different groups. Black arrows denote the basement membrane of the glomerulus, and yellow arrows indicate glomerular fibrosis. Data are expressed as means ± SEM, *n* = 8. * *p* < 0.05 compared with control.

In order to assess the impact of MP exposure on the structure of the kidney tissue in mice, we performed periodic acid–Schiff (PAS) staining on the kidney sections. As shown in Figure 2A,C, the mice treated with MPs displayed larger glomerular tuft areas than the control mice. Specifically, the area was increased by 13% and 19% in the L-MPs and H-MPs groups of mice, respectively. In addition, the renal fibrosis presented in the blue area, assessed via Masson’s trichrome staining, was not altered by MP exposure (Figure 2B,D). These findings reveal that PS-MP exposure at environmentally relevant doses altered the histological structures of the mice kidneys.

### 2.3. Effects of PS-MP Exposure on Kidney Transcriptome

**Figure 3:**
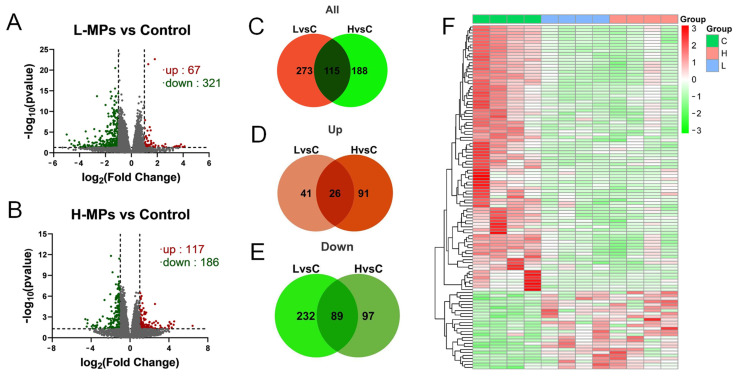
Distributions of DEGs in renal cortex after exposure to PS-MPs. Volcano plot of DEGs in the L-MPs group (**A**) and H-MPs group (**B**) compared to the control group. Venn diagram of all DEGs (**C**), up-regulated genes (**D**), and down-regulated genes (**E**) in the L-MPs and H-MPs groups. Heat map of common DEGs (**F**) in both the L-MPs and H-MPs groups. L vs. C, L-MPs vs. Control; H vs. C, H-MPs vs. Control.

Investigating the transcriptome can help to illustrate the molecular alterations brought about by certain chemical compounds. To investigate the responses of the kidneys to MP exposure, the renal cortex transcriptome was analyzed and differentially expressed genes (DEGs) were identified. The mice in L-MPs group showed significant changes in 388 renal genes (67 up-regulated and 321 down-regulated, Figure 3A) compared to controls, while 303 genes (117 up-regulated and 186 down-regulated, Figure 3B) were altered in mice subjected to high doses of MPs. In addition, 26 genes were up-regulated both in the L-MPs and H-MPs groups compared to the control (Figure 3D), and 89 genes were down-regulated in both groups (Figure 3E). The 115 genes commonly changed in the renal cortex of mice in both the L-MPs and H-MPs groups are clustered in the heat map shown in Figure 3F.

### 2.4. Gene Ontology (GO) and Kyoto Encyclopedia of Genes and Genomes (KEGG) Analysis of Enrichment of Differentially Expressed Genes

**Figure 4:**
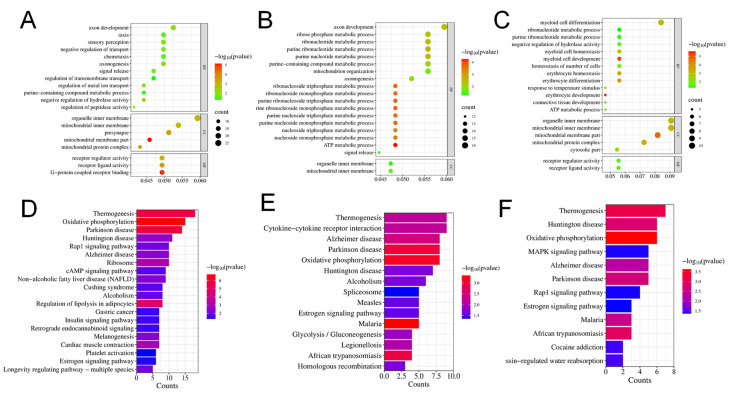
Effects of PS-MPs on the transcriptome of mice kidneys. Top 20 GO items for DEGs shared between the L-MPs group (**A**) or H-MPs (**B**) group and the control group. Top 20 GO items for common DEGs in the L-MPs and H-MPs groups (**C**). Significant KEGG pathways for DEGs between the L-MPs group (**D**) or H-MPs (**E**) group and the control group. Significant KEGG pathways for common DEGs in the L-MPs and H-MPs groups (**F**).

The differentially expressed genes were categorized into three primary GO categories: biological process (BP), cellular component (CC), and molecular function (MF). The DEGs were considerably distributed in 358 GO items, including 241 BP items, 61 CC items, and 56 MF items when comparing the L-MPs group and control group. In the comparison between the H-MPs group with the control group, 248 DEGs were distributed across 241 BP items, 177 CC items, and 33 MF items. The top 20 enriched GO items were summarized in Figure 4A,B. To better understand the effects of PS-MPs, we further conducted enrichment analysis on the common DEGs of the L-MPs and H-MPs groups. The 115 DEGs were distributed in 156 GO items, including 98 BP items, 35 CC items, and 23 MF items (Figure 4C), and the top five enriched GO terms were the mitochondrial inner membrane, organelle inner membrane, myeloid cell differentiation, mitochondrial membrane part, and mitochondrial protein complex.

Subsequently, KEGG pathway annotation was performed to gain a deeper understanding of the biological pathways that were altered due to MP exposure. In the L-MPs group, DEGs were enriched in 29 pathways (Figure 4D), whereas 16 pathways were enriched by DEGs between the H-MPs group and control group (Figure 4E). In addition, the common DEGs of the two groups were mainly enriched in pathways pertaining to thermogenesis, Huntington’s disease, oxidative phosphorylation, mitogen-activated protein kinase (MAPK) signaling pathway, Alzheimer’s disease, etc. (Figure 4F).

### 2.5. Protein–Protein Interaction (PPI) Network Analysis and Hub Gene Identification

**Figure 5:**
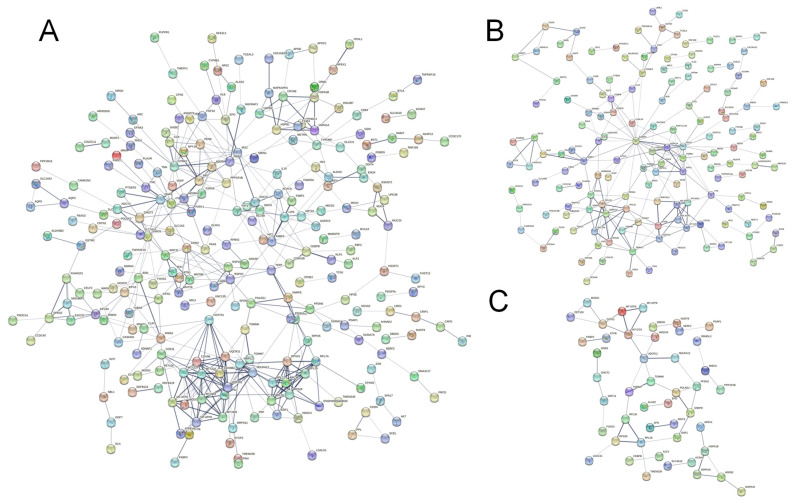
Effects of PS-MPs on PPI network and hub genes. PPI network of the L-MPs group (**A**) and H-MPs group (**B**) compared with the control group and the common DEGs of the groups (**C**). The nodes represent proteins, while the edges represent the number of interactions. The color saturation of the edges represents a functional association’s confidence score. The node degree indicates the number of typical interactions per node. Only interactions with a high confidence score (0.4) are displayed; disconnected nodes have been concealed.

To investigate the important role of protein interactions in the PS-MPs group, we performed a PPI network analysis using the STRING online database and calculated the correlation degree of each protein. The PPI network in the L-MPs group involved 338 nodes and 452 edges, whereas 229 nodes and 225 edges were involved in the H-MPs group, and the common DEGs of two groups had 94 nodes and 45 edges. Figure 5A summarizes the correlation results for the DEGs associated with the L-MPs group and the control group. The top 10 proteins were neurotrophic receptor tyrosine kinase 2 (NTRK2), cytochrome b-c1 complex subunit 8 (UQCRQ), cytochrome b-c1 complex subunit 10 (UQCR11), cytochrome c oxidase subunit 2 (MT-CO2), mitochondrial ribosomal protein L22 (MRPL22), cytochrome c oxidase subunit 3 (MT-CO3), cytochrome c oxidase subunit 6B2 (COX6B2), ribosomal protein S28 (RPS28), cytochrome c oxidase subunit 7A1 (COX7A1), and cytochrome c oxidase subunit 5B (COX5B) (Figure 5A), which were located in the core of the network and linked to many other DEGs. The top 10 proteins for the H-MPs group and the control group were glyceraldehyde-3-phosphate dehydrogenase (GAPDH), alpha-synuclein (SNCA), small nuclear ribonucleoprotein D2 polypeptide (SNRPD2), mitochondrial import inner membrane translocase subunit Tim13 (TIMM13), heat shock protein beta-2 (HSPB2), UQCR11, MT-CO3, ATP synthase subunit epsilon (ATP5E), heat shock protein family A member 1B (HSPA1B), and histone H2AX (H2AFX) (Figure 5B). We also analyzed the potential interaction network of common DEGs of two comparison groups. The top 10 proteins of the common genes were UQCR11, MT-CO3, 60S ribosomal protein L28 (RPL28), cytochrome c oxidase assembly protein COX11 (COX11), mitochondrial import receptor subunit TOM7 homolog (TOMM7), 60S ribosomal protein L35 (RPL35), mitochondrial import receptor subunit TOM6 homolog (TOMM6), DNA-directed RNA polymerase II subunit RPB11-a (POLR2J), prefoldin subunit 1 (PFDN1), and RPS28 (Figure 5C). In addition, the most prominent protein was NTRK2 in the L-MPs group, with a degree of 16, while GAPDH was the most prominent protein in the H-MPs group, with a degree of 26, and UQCR11 was found in both groups, with degree of 5, respectively.

## 3. Discussion

As an important metabolic and excretory organ, the anatomical and physiological characteristics of the kidney determine its susceptibility to MP exposure. In this study, we exposed mice in a mouse model exposed to MPs through drinking water and evaluated the blood biochemical indicators related to renal function, renal histopathology, and transcriptomic responses in the renal cortex. The main results of our study are as follows: (1) PS-MPs lowered BUN levels; (2) PS-MPs significantly increased glomerular tuft area; (3) PS-MPs disturbed the renal cortical transcriptome, causing abnormalities in thermogenesis and mitochondrial oxidative phosphorylation pathways.

The kidney is vital for maintaining homeostasis in mammals, which plays an important role in metabolic waste excretion, acid–base balance, electrolyte balance regulation, and endocrine regulation. BUN and serum CRE are commonly used as biomarkers to evaluate renal function, and high levels of BUN and serum CRE suggest inhibited glomerular filtration function [27]. In the present study, we found that exposure to both low (0.1 mg/L) and high (1 mg/L) environmental doses of MPs reduced BUN levels in mice, implying an increase in glomerular filtration efficiency. Although no significant changes in serum CRE were observed in the MP-exposed mice in our study, there was a decrease trend in the H-MPs group. In addition, we observed an increase in glomerular tuft area in the kidney tissue of the MP-exposed mice, which is also suggestive of an increase in glomerular filtration at the histopathological level. Contrary to our findings, Meng et al. reported increases in BUN levels in mice after exposure to a higher dose (5 mg/day) of MPs via gavage for four weeks [21]. It can be inferred from these results that exposure to lower doses of MPs can induce compensatory glomerular hypertrophy, enhance filtration performance, and boost BUN clearance in mice. However, as the dose of MP exposure increases, the kidney’s compensatory capacity may be exceeded, resulting in impaired renal filtration function. Another noteworthy finding in our study was that exposure to MPs did not result in aberrant renal fibrosis. However, a study conducted by Shi et al. found that exposure to 1 mg/L MPs for 180 days led to renal tubular cytoplasmic vacuolation, lipid drops, and collagen fiber accumulation, which eventually promoted the incidence of kidney injury in the mice under study [28]. These results suggest that the hazards of MPs at higher concentrations and longer exposure times require additional attention.

The kidney, as one of the organs with the highest levels of energy consumption in the body, is densely packed with mitochondria [29]. ATP produced by mitochondria via oxidative phosphorylation serves as the main energy source for kidney. Numerous studies have demonstrated that mitochondrial function is impaired in various causes of acute kidney injury and chronic kidney disease [29,30]. Therefore, researchers have proposed that mitochondria could be a target for the prevention and treatment of kidney damage caused by harmful factors [31,32]. An in vitro investigation has shown that a high dose (0.8 mg/mL) of PS-MP exposure could cause mitochondrial dysfunction in human kidney proximal tubular epithelial cells (HK-2 cells) [22]. The results regarding the renal cortical transcriptome in our study revealed that the expression of genes related to the mitochondria were significantly up-regulated in both the L-MPs group and H-MPs groups, and most genes were enriched in thermogenesis and oxidative phosphorylation pathways. We suggest that this alteration could be the molecular basis for the compensation of renal structure and function induced by environmentally relevant doses of MP exposure. It should be noted that as the MP exposure dose increases, kidney mitochondria may become dysfunctional, leading to renal decompensation and exacerbating the nephrotoxicity of MPs. UQCR11 and MT-CO3, which were both up-regulated, were identified as common hub genes for MP nephrotoxicity using PPI analysis. UQCR11 belongs to the mitochondrial respiratory chain and is a component of the panthenol–cytochrome c reductase complex. Han et al. verified that UQCR11 plays a crucial role in patulin-induced nephrotoxicity, exerting influences on apoptosis, oxidative phosphorylation, ribosome, cell cycle, and nucleotide metabolism in kidney cells [33]. MT-CO3 is a cytochromic c oxidase subunit III gene whose mutation may alter the kinetics of cytochrome c oxidase, an enzyme that enhances oxygen transport during oxidative phosphorylation [34,35]. In summary, our study implies that mitochondrial dysfunction might be involved in MP-induced kidney impairment, affecting the process of oxidative phosphorylation.

Our study provides information on the changes in the structure and gene expression profile of the mouse renal cortex following MP exposure, but it has some limitations. Firstly, the particle sizes of the PS-MPs employed in published studies vary a lot [22,25,36], whereas 1 μm PS-MPs were utilized in the current investigation. Although MPs with tiny particle sizes, particularly those on a nano diameter scale, are more likely to be deposited in the kidney [36], it remains to be established whether MPs with smaller particle sizes induce more severe renal structural and transcriptome abnormalities. In addition, the exposure duration in this study was only 8 weeks, which is far shorter than the average human lifetime exposure. The reported changes in the renal structure and transcriptome may not fully reflect the renal toxicity after long-term exposure to MPs. Thus, the effects of long-term exposure to MPs, especially lifelong exposure, on the kidney are yet to be revealed.

## 4. Materials and Methods

### 4.1. Materials

Monodisperse PS-MPs, 1 μm in diameter, were provided by Tianjin Baseline Chromatography Technology Development Center (Tianjin, China). The PS-MPs stock solution (25 mg/L) was stored at 4 °C. Before usage, the microspheres were entirely re-dispersed using sonication and diluted into the desired concentration.

### 4.2. Mice and Treatments

Six-week-old male C57BL/6 mice were ordered from the Beijing Vital River Laboratory Animal Technology Co., Ltd. (Beijing, China). All mice were kept in an environment with a controlled temperature (22 ± 2 °C) and humidity (50 ± 5%) with free access to normal food and water. After acclimatization, 24 mice were randomly assigned to one of three groups (*n* = 8 for each group): control, L-MPs, or H-MPs. The mice in the L-MPs and H-MPs groups were given sterilized drinking water with 0.1 mg/L or 1 mg/L of MPs, respectively, whereas the animals in the control group were given sterilized water alone. Based on previous studies, 0.1 and 1 mg/L were selected as environmentally relevant doses [25] (Xiong et al., 2023). The mice were euthanized after 8 weeks of exposure. The serum was collected and frozen at −80 °C for subsequent biochemical analysis. The kidney samples were weighed and frozen for further examination or fixation for histopathology. This study’s animal experimental methods were approved by the Institutional Animal Care and Use Committee of Zhejiang Chinese Medical University. The kidney coefficient was calculated as the ratio of kidney weight (wet weight) to body weight.

### 4.3. Routine Kidney Function Blood Test

To test the kidney function of mice, the levels of BUN, CRE, and UA in the serum were measured using a Hitachi 3100 Automatic Analyzer (Hitachi High-Tech Science Systems Inc., Tokyo, Japan) in the Laboratory Animal Center of Zhejiang Chinese Medical University.

### 4.4. Kidney Histopathology

The kidney tissues were fixed in 4% paraformaldehyde (PFA) solution, paraffin-embedded, and sectioned. To observe the structural changes, the kidney sections (5 μm thickness) were stained via PAS reaction. Renal PAS staining showed that the basement membrane, mesangial matrix, glycogen, and glycoprotein were purplish red, and the nucleus was blue, which was conducive to the observation of glomerular morphology. A minimum of 20 glomerulus were randomly selected for each kidney slide, and images were captured using the Leica DM6B microscope (operated by a researcher in a blind procedure). Another researcher blindly assessed the individual glomerular tuft area by using ImageJ 1.51. Additionally, Masson’s trichrome staining was used to identify fibrotic regions in the kidney sections. Masson staining revealed that the basement membrane, mesangial matrix, and collagen were blue, whereas the immune complex and blood cells were red, and the nucleus was blue-black. Researchers blindly and randomly captured the Masson staining images. The collagen deposition was also quantitatively measured using ImageJ software.

### 4.5. RNA Extraction and Sequencing

TRIzol reagent was used to extract total RNA from the renal cortices of the mice in the three groups. RNA integrity and quality were assessed using an Agilent 2100 bioanalyzer (Agilent Technologies, Santa Clara, CA, USA). The mRNA was enriched by Oligo (dT) magnetic beads and fragmented by an interrupting reagent. The fragmented RNA was used as a template to synthesize cDNA. After being end-repaired, A-tailed, and attached to sequencing junctions, cDNAs of 370~420 bp were screened with the AMPure XP system (Beckman Coulter, Beverly, MA, USA). PCR products were amplified and purified using AMPure XP beads to generate the library. The quality of the library was evaluated and then sequenced on an Illumina NovaSeq 6000.

### 4.6. Sequencing Data Analysis

Sequenced fragments were converted into reads via CASAVA base identification of the image data from the high-throughput sequencer. The raw data were filtered by erasing reads with adapters or with N (N indicates that the base information cannot be determined) and discarding low-quality reads (the number of bases with Qphred ≤ 20 accounts for more than 50% of the total read length). The reference genome was indexed using Hisat2 (version 2.0.5), and the number of reads was calculated using the Counts feature. The statistically significant DEGs were identified via differential gene expression analysis, which was carried out using DESeq2 R package (version 1.20.0) with a threshold of *p* ≤ 0.05 and |log_2_(fold change)| ≥ 1.

### 4.7. Enrichment Analysis of Differentially Expressed Genes

The clusterProfiler R package (version 3.8.1) was applied to perform GO and KEGG enrichment analysis. The differentially expressed genes were deemed strongly enriched by GO terms with a corrected *p* ≤ 0.05. KEGG is a database resource used for deducing the high-level function and utility of biological systems such as cells, organisms, and ecosystems from molecular-level data. The top 20 items or pathways with *p* < 0.05 were selected to draw the bubble map and bar map.

### 4.8. PPI Analysis of Differentially Expressed Genes

The STRING database (https://stringdb.org, accessed on 13 April 2023) was used to analyze the PPI networks between differentially expressed genes, including the direct and indirect associations of proteins. The network was built based on known interactions of the selected reference species presented in the database. The results of our PPI analysis revealed expression change information for each differentially expressed gene.

### 4.9. Statistical Analysis

The results of the experiments are shown as the mean ± SEM. A one-way analysis of variance (ANOVA) followed by either Dunnett’s test or Student’s *t*-test was used to compare the exposed mice to the controls. The significance threshold was set at *p* < 0.05.

## 5. Conclusions

In summary, the results of our investigation show that exposure to environmentally relevant doses of MPs through drinking water changed the renal structure and filtration function in mice, and this was mainly manifested by an increase in the glomerular tuft area and reduction in BUN levels. Our transcriptomic findings indicate that mitochondrial functional genes were most sensitive to MP exposure and that thermogenesis- and oxidative phosphorylation-related pathways might be potential targets for nephrotoxicity of MPs.

## Data Availability

Data are available upon reasonable request.
